# Extracellular vesicles in malignant and normal B lymphocyte growth and development

**DOI:** 10.20517/evcna.2025.42

**Published:** 2025-12-10

**Authors:** Kaitlyn E. Mayne, Rashid Jafardoust, Hong-Dien Phan, Sherri L. Christian

**Affiliations:** ^1^Department of Biochemistry, Memorial University, St. Johns A1C 5S7, Canada.; ^2^Beatrice Hunter Cancer Research Institute, Halifax B3H 4R2, Canada.

**Keywords:** Extracellular vesicles, B lymphocyte, normal development, cancer development, microenvironment

## Abstract

Extracellular vesicles (EVs) are central mediators of intercellular communication in both healthy and malignant states. In normal B lymphocyte (cell) biology, EVs derived from B cells, mast cells, T cells, and mesenchymal stromal cells regulate maturation, antigen presentation, and activation. B cell-derived EVs can either suppress excessive activation to maintain immune homeostasis or amplify responses during an active immune response. Modulation of these responses often occurs via phosphoinositide 3-kinase signaling pathways in recipient cells. In B cell malignancies, such as leukemias, lymphomas, and multiple myeloma, EVs play pivotal roles in disease progression and therapy resistance. Tumor- and stromal-derived EVs can transfer pro-survival proteins, regulatory RNAs, and drug-resistance factors to directly promote tumor progression. In addition, EVs can shape the tumor microenvironment to indirectly promote tumor progression through macrophage polarization, stromal cell reprogramming, and suppression of anti-tumor immunity. Conversely, under certain conditions, B cell EVs can enhance immune surveillance by stimulating T cells and presenting antigen, highlighting their dual potential in cancer biology. Clinically, B cell-derived EVs represent promising liquid biopsy biomarkers: increases in EV abundance, expression of surface antigens, altered protein cargo, and distinct RNA signatures have been associated with disease stage, treatment response, and patient outcomes. Despite this potential, variability in EV isolation and analysis methods remains a barrier to clinical translation. Moving forward, identifying robust biomarker signatures across platforms and clarifying mechanisms of cargo selection and EV uptake will be critical for advancing diagnostic and therapeutic applications. Overall, B cell-derived EVs act as contextual regulators of immune function and malignancy, positioning them as both modulators of disease progression and promising clinical tools.

## INTRODUCTION TO EXTRACELLULAR VESICLES (EVs)

Extracellular vesicles (EVs) are a heterogeneous group of phospholipid bilayer-enclosed particles that contain cargo reflecting the cell of origin, including transmembrane proteins and cytoplasmic components, such as cytosolic proteins, DNAs, messenger RNA (mRNAs), and microRNAs (miRNAs)^[1]^. Cells can secrete different types of EVs, which are classified based on their release pathway^[2]^. Exosomes originate from intraluminal vesicles (ILVs) that form through the inward budding of the endosomal membrane, resulting in the formation of multivesicular bodies (MVBs). This is followed by secretion upon fusion of the MVB with the cell plasma membrane. Exosomes typically range in size from 30 to 150 nm. Slightly larger but overlapping in size, ectosomes, also known as microvesicles, are generally considered to be 100 nm to 1 µm in size and formed by direct outward budding from the surface of the plasma membrane. The largest type of EVs are apoptotic bodies generated by cells undergoing the final stages of apoptosis, which can range from 1 µm to 5 µm. Other EVs have also been described, such as apoptotic exosome-like vesicles (ApoExos), which are similar in size to exosomes but are produced by different biogenesis processes^[3,4]^, and oncosomes, which are large EVs that bleb from the plasma membrane of solid tumors^[5]^.

There are currently no established techniques for definitively classifying the various kinds of vesicles so researchers must consider a number of complementary techniques in addition to size, as outlined in the minimal information for studies of extracellular vesicles (MISEV) guidelines^[6]^. Unless the authors have specifically proven that they are analyzing exosomes or ectosomes, we will use the term EV in this review.

EVs are key players in cell-cell communication. EVs released into the extracellular space are key communicators between normal and malignant cells throughout the body. They can transfer bioactive molecules to, or interact with, neighboring or distant cells through endocytosis^[7]^, fusion^[8]^, or receptor-ligand interactions^[9]^. Following these interactions, EVs shuttle functional lipids, nucleic acids, or proteins that can alter the biological functions of target cells, thereby contributing to both physiological and pathological processes^[1,10-12]^. Generally, EVs have the potential to provide combinatorial information to multiple cells in their microenvironment and throughout the body.

## THE ROLE OF EVs IN B CELL DEVELOPMENT AND ACTIVATION

**Figure 1:**
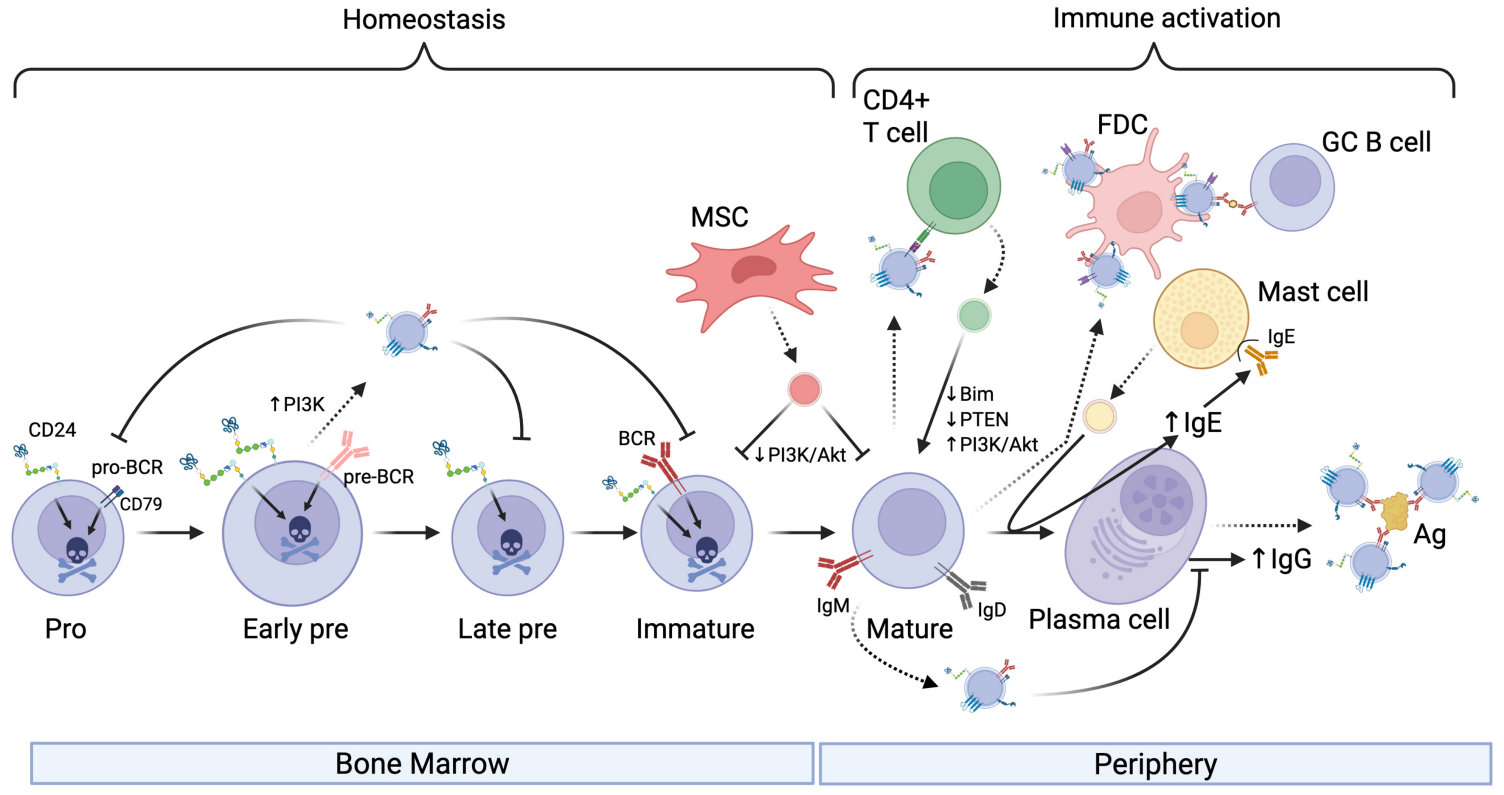
The influence of EVs on B cell development and activation. EVs (shown as small vesicles colored according to the donor cell) secreted by developing B cells can affect B cell homeostasis by transferring pro-apoptotic receptors to recipient B cells and can limit class-switching. In addition, EVs secreted by MSCs can limit B cell survival by downregulating PI3K signaling in bone marrow-derived or circulating B cells. EVs secreted by B cells can affect T cell activation directly via MHC-mediated presentation of peptide to T cells or via decorating FDC. Moreover, EVs released by Mast cells can promote antibody class-switching to IgE while T cell-derived EVs can upregulate PI3K pathway activation to increase secretion of IgG. EVs secreted by antibody-producing cells, such as plasma cells shown here, can neutralize Ag in circulation. Activation is indicated by arrowheads, and inhibition is indicated by perpendicular lines. Dashed lines indicate EV release from donor cells. Created in BioRender. Christian, S. (2025) https://BioRender.com/3607mjk. EVs: Extracellular vesicles; MSCs: mesenchymal stromal cells; PI3K: phosphoinositide 3-kinase; Akt: protein kinase B; MHC: major histocompatibility complex; FDC: follicular dendritic cell; Ag: antigen; BCR: B cell receptor; pro-BCR: pro-B cell receptor; GC B cell: germinal center B cell; PTEN: phosphatase and tensin homolog; Bim: Bcl-2-like protein 11.

Hematopoietic stem cells differentiate into all types of blood cells, including B cells, in the bone marrow^[13]^. One of the earliest B cell precursors is the pro-B cell, characterized by the expression of cluster of differentiation (CD)19 and CD43, among others. The recombination of V, D, and J gene segments in pro-B cells leads to the assembly of functional immunoglobulin (Ig) heavy chain genes and differentiation into pre-B cells. These cells are characterized by expressing the pre-B cell receptor (pre-BCR). They then develop into immature B cells, which express functional IgM and IgD on their surface in the context of the B cell receptor (BCR). Finally, mature B cells are released from bone marrow to the circulation, where they can be activated in response to antigen and T cell help [Figure 1].

### B cell-derived EVs influence B cells

B cell-derived EVs have been shown to act as mediators in immune regulation, influencing immune cell activity through antigen presentation, immune cell activation, and suppression [Table 1]. Raposo *et al*. demonstrated a foundational role for B cell-derived EVs, showing that they can present antigens directly to T cells^[9]^. These EVs carry major histocompatibility complex (MHC) class II molecules and their associated peptides, which are required for T cell activation. Subsequent studies reinforced this view, for example, B cell-derived exosomes can induce T_H_2-type immune responses by presenting allergenic peptides^[34]^. Follicular dendritic cells (FDCs) support the presentation of antigen to B cells in lymphoid follicles. FDCs do not internalize and process antigens; rather, they bind EVs on their cell surface^[14]^. These data suggest that FDC-bound EVs may function to optimize B cell selection, proliferation, isotype switching, and memory B cell differentiation in the germinal center (GC). The function of MHC class II on FDC-bound EVs is unclear as normally T cells do not interact with FDCs^[35]^. Overall, there is clear evidence that B cell-derived EVs influence regulation of B cell development and activation during an active immune response.

**Table 1:** Effects of EVs on normal and malignant B cells

**EV source** → **Target**	**Effect on normal cells**	**Effect on malignant B cells**
Effect of normal B cell EVs on normal immune cells		
B cell EVs from GC → coat follicular dendritic cells	Enhance B cell development and activation in GC^[14]^	N/A^a^
B cell EVs → B cells	Transfer miR-5099 and lncRNA Gm26917 to suppress CSR^[15]^	N/A
B cell EVs → T cells	Triggers inflammatory CD4^+^ T cell response via IL-2 secretion^[9]^	N/A
B cell-derived allergen-pulsed EVs → T cells	Induce CD4^+^ T cell proliferation and secretion of Th2 cytokines^[16]^	N/A
Effect of normal immune cell EVs on normal B cells or B cell malignancies		
B cell EVs → B cells	B cells stimulated by CD24 or IgM transfer functional pro-apoptotic receptors^[17]^	Malignant B cells exchange ectosomes in response to CD24 stimulation. May promote apoptosis^[17,18]^
T cell EVs → B cells	Deliver miR-155-3p, miR-25-3p, miR-20a-5p to enhance proliferation, class-switching, and survival. Secretion regulated by Rab27a^[19]^ Increase transfer of ceramide to EVs to promote IgG production, CSR, and B cell survival via PI3K^[20]^	N/D^a^
BMSC Evs → B cells	EVs from BMSC primed with inflammatory cytokines suppress B cell activation^[21]^	Promote MM cell growth, migration, and drug resistance^[22]^
Effect of malignant B cell EVs on other cells		
CLL EVs → T cells	Promote T cell exhaustion to decrease anti-tumor immunity^[23]^	N/D
DLBCL EVs → T cells	Cause Th2 cells to upregulate PD-1 or undergo apoptosis^[24]^	N/D
DLBCL EVs → macrophage	Increase M2 tumor-associated macrophages to support tumor progression and survival^[25]^	N/D
CLL EVs → vascular endothelial cells	Cause increased secretion of IL-6 into the TME to enhance CLL survival^[26]^	N/D
CLL EVs → Stromal cells	Induce a pro-inflammatory, CAF phenotype and boot angiogenesis to promote CLL progression^[27]^	N/D
MM EVs →MSC	Inhibit osteoblastic differentiation of MSCs and induce high levels of IL-6 in the TME to promote proliferation and migration of MM cells^[28]^	N/D
DLBCL EVs → DLBCL	Downregulate CD20 expression via the transfer of miR-125-5p^[29]^ Chemo resistant DLBCL secrete EVs with high levels of CA1 to upregulate survival pathways^[30]^	N/D
Effect of solid tumor EVs on B cells		
Myeloid-derived suppressor cell EVs from glioblastomas → B cells	Induce the generation of Breg cells to suppress anti-tumor immunity^[31]^	N/D
Esophageal squamous cell carcinoma EVs → B cells	Induce the generation of Breg cells to suppress anti-tumor immunity^[32]^	N/D
Hepatocellular carcinoma → B cells	Induce the generation of Breg cells to suppress anti-tumor immunity^[33]^	N/D

^a^N/A: Not applicable, N/D: not determined; BMSC: bone marrow stromal cell; CAF: cancer-associated fibroblast; CD: cluster of differentiation; CLL: chronic lymphocytic leukemia; CSR: class-switch recombination; CA1: carbonic anhydrase 1; DLBCL: diffuse large B-cell lymphoma; EC: endothelial cell; EVs: extracellular vesicles; FasL: Fas ligand; GC: germinal centre; IgG: immunoglobulin G; IgM: immunoglobulin M; IL: interleukin; lncRNA: long non-coding RNA; M2: M2 macrophage; MDSC: myeloid-derived suppressor cell; miR/miRNA: microRNA; MM: multiple myeloma; MSC: mesenchymal stromal cell; PD-1: programmed cell death protein 1; TME: tumor microenvironment; Treg: regulatory T; Th2: T helper 2.

Stimulation of B cells via various surface receptors has been shown to increase EV release. CD24 is a surface marker that is increased in the pre-B cell stage and promotes apoptosis in developing B cells^[36]^. In response to antibody-mediated engagement of CD24 or the BCR, we found that B cells release more EVs that carry CD24, the BCR, as well as phosphatidylserine on their surface^[17,18,37]^. These EVs also facilitate the exchange of surface CD24 and the BCR between different B cell populations^[17]^. Notably, the transferred CD24 and BCR are functional on the recipient cells, endowing them with increased sensitivity to these pro-apoptotic stimuli^[17]^. This transfer of pro-apoptotic receptors may result in homeostatic regulation by inducing apoptosis of bystander cells in response to activation of a subset of cells, potentially in response to increased damage-associated molecular pattern (DAMP) levels in the case of CD24^[38]^ or autoimmune antigens in the case of the BCR^[39]^. Both increased DAMPs and autoimmune recognition could result in pathology so limiting B cell survival at this stage may be beneficial.

Splenic B cells increase the release of EVs expressing IgG derived from the cell surface in response to stimulation of CD40 and interleukin (IL)-4^[40]^. Similarly, using a CD63-Cre-emGFP model, others have found that B cells that have been stimulated to class-switch via lipopolysaccharide (LPS) and IL-4 treatment secrete IgG-positive EVs, which end up in circulation^[41]^. These IgG-bearing EVs can effectively neutralize influenza virus, demonstrating their functionality. IgM has also been reported to be carried on and inside EVs; however, the relevance of this observation is unclear as the binding of antigen by these EVs was not analyzed statistically or in an infection model^[42]^. Regardless, these observations suggest that the normal immune response may include both circulating, free antibodies and EV-bound antibodies, which may function with different efficacy or avidity to neutralize antigens.

### Mast cell & mesenchymal stromal cell (MSC)-derived EVs

EVs derived from Mast cells can influence B-cell maturation after antigen-mediated activation. Mast cell-derived EVs from RBL-2H3 cells contain bioactive lipids such as prostaglandins, including Prostaglandin E2 (PGE2), which could potentially regulate isotype switching and plasma cell differentiation to promote IgE secretion^[43-45]^. This would serve as a positive feedback loop whereby mast cells would promote their own sensitization by increasing the levels of circulating IgE.

EVs from Mesenchymal stromal cells (MSCs) and primary B cells can limit excessive B cell activation^[21]^. EVs, particularly from MSCs primed with inflammatory cytokines, can suppress phosphorylation of Akt and downstream effectors such as Glycogen Synthase Kinase-3β (GSK-3β) and p60 S6-Kinase via the EV-mediated transfer of miR-155-5p. The same EVs prevented the re-organization of the actin cytoskeleton in response to BCR stimulation, a key process in B cell activation. Additionally, exchange of EVs between primary B cells that are enriched in miR-5099 acts as a class-switch recombination (CSR) suppressor by targeting the RNA-binding protein polypyrimidine tract binding protein 3 (PTPB3), a known cofactor for activation-induced deaminase (AID)-mediated isotype switching^[15]^. Conversely, the long non-coding RNA (lncRNA) GM26917, found in CH12F3 lymphoma cell-derived EVs, enhances CSR by antagonizing miR-5099 activity^[15]^. Thus, EVs from normal, healthy cells tend to suppress B cell activation, which limits excessive proliferation and cytokine secretion, whereas lymphoma cells tend to promote activation, an observation that will be expanded on below.

### T cell-derived EVs

During T cell-dependent B cell activation, T cells release EVs into the extracellular space within the immunological synapse. B cells take up EVs through this interaction, which facilitates the transfer of miRNA-335 and microRNA-92 (miR-92)^[46]^. Transfer of miRNA-335 via this route was associated with the downregulation of SOX4^[46]^, which is required for the survival of pro B cells^[47]^ but has an unknown function in B activation. During synapse formation, EVs derived from CD4+ T cells, enriched in miR-20a-5p, miR-25-3p, and miR-155-3p, facilitate the transfer of regulatory miRNAs to B cells in the GC^[19]^. These miRNAs shape B cell populations by downregulating pro-apoptotic and inhibitory genes such as BIM and PTEN, which regulate apoptosis and cell survival, respectively. This transfer indirectly promotes B cell proliferation, CSR, survival, and antibody production. Alternatively, disruption of T cell EV release by Rab27a silencing or pharmacological inhibition leads to impaired GC formation and a significant reduction in serum IgG levels *in vivo*^[19]^.

T cell-derived EVs can also affect B cell activation in a non-synaptic manner. Pyruvate kinase M2 (PKM2) is a key enzyme in glycolysis that is expressed by T cells^[48]^. When the expression of PKM2 was increased in T cells by hyperhomocysteinemia, there was an increase in the transfer of ceramide to B cells via EVs^[20]^. This transfer promotes the production of IgG from B cells. In contrast, the lack of PKM2 was associated with decreased IgG production and suppression of the phosphoinositide 3-kinase (PI3K)/Akt pathway, suggesting that T cell-derived EVs actively regulate the survival of recipient B cells. Thus, in the context of an immunological activation signal, EVs tend to promote B cell activation and differentiation, potentially via activation of PI3K signalling pathways.

Interestingly, we recently found that the PI3K/mTORC2 pathway, which is well known for promoting cell survival and proliferation in normal and malignant B cells^[49]^, is also required for EV release in response to CD24 stimulation^[50]^. As noted above, PI3K is also activated by EVs released by T cells^[19]^ and inhibited by EVs released by MSC^[21]^. Thus, it is also possible that these EVs could promote or prevent EV release by the recipient B cells where EVs taken up by recipient cells shape subsequent release of EVs.

Overall, these data suggest that in situations of normal B cell homeostasis or when the EVs come from primary B cells, they tend to suppress activation and proliferation. In contrast, in the case of an active immune response, EVs form part of a positive feedback loop that includes Mast cells and T cells to further enhance B cell activation and the immune response.

## EVs IN TUMOR PROGRESSION AND SURVIVAL

B cell cancers comprise leukemias and lymphomas. Leukemias originate in the bone marrow and circulate in the bloodstream while lymphomas form in the lymph nodes or spleen and generally circulate in the lymphatic system. Multiple myeloma (MM) develops from plasma cells in the bone marrow.

**Figure 2:**
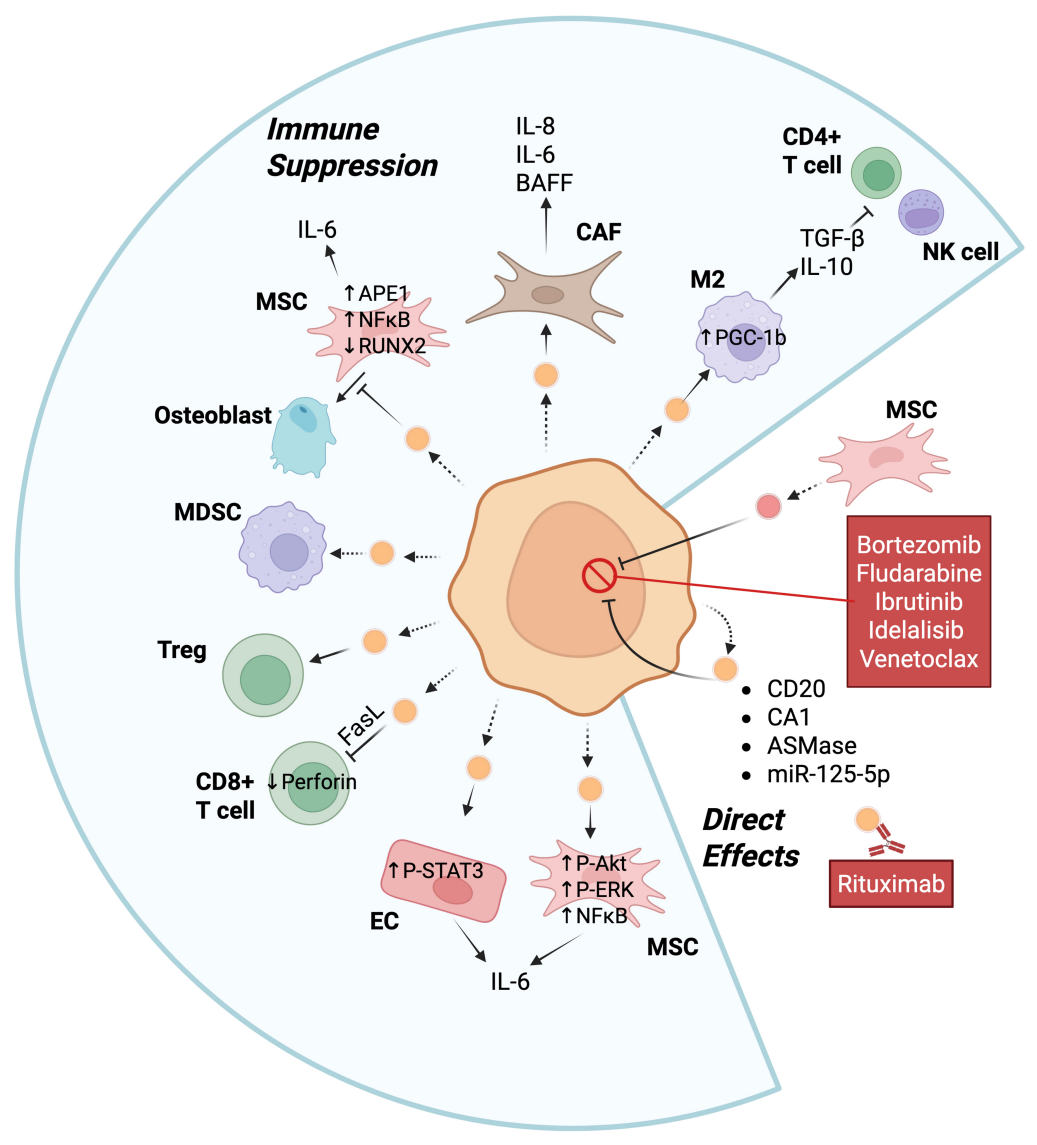
EVs can promote tumor progression of B cell malignancies through multiple mechanisms. The central orange cell represents a malignant B cell (e.g., MM, CLL, or DLBCL), and the small orange vesicles are the EVs released by the malignant B cell. These EVs can regulate T cell activation, osteoblast differentiation, the transformation of EC into CAF, the polarization of macrophages to M2, as well as be exchanged between cancer cells. MSCs can release EVs that directly affect B cell malignancies or take up EVs from B cell cancers. Activation of either MDSC or regulatory T cells can also suppress immune activation. Examples of intracellular mediators and extracellular cytokine release are shown. Bullets indicate proteins known to be transferred by EVs to recipient cells. Dashed arrows indicate the release of EVs by cells while solid arrows indicate the interaction of an EV with a target cell or release of a cytokine. Created in BioRender. Christian, S. (2025) https://BioRender.com/53cq8kd. Ag: Antigen; Akt: protein kinase B; APE1: apurinic/apyrimidinic endonuclease 1; ASmase: acid sphingomyelinase; BAFF: B-cell activating factor; CAF: cancer-associated fibroblast; CA1: carbonic anhydrase 1; CLL: chronic lymphocytic leukemia; DLBCL: diffuse large B-cell lymphoma; EC: endothelial cell; EVs: extracellular vesicles; FasL: Fas ligand; IL: interleukin; M2: M2 macrophages; MDSC: myeloid-derived suppressor cell; miR-125-5p: microRNA-125-5p; MM: multiple myeloma; MSCs: mesenchymal stromal cells; NFkB: nuclear factor kappa-light-chain-enhancer of activated B cells; NK cell: natural killer cell; P-Akt: phosphorylated Akt; P-ERK: phosphorylated extracellular signal-regulated kinase; PGC-1β: peroxisome proliferator-activated receptor γ coactivator 1β; P-STAT3: phosphorylated signal transducer and activator of transcription 3; RUNX2: runt-related transcription factor 2; TGF-β: transforming growth factor-β; Treg: regulatory T.

Tumor-derived EVs carry proteins and nucleic acids that play a role in mediating crosstalk with the tumor microenvironment and can promote tumor progression through multiple mechanisms directly or indirectly [Table 1 and Figure 2].

### EVs directly support tumor progression

EVs from B cell malignancies have been shown to directly promote cancer cell survival by transferring proteins and regulatory RNAs to recipient cells. EV-mediated transfer of miRNAs between diffuse large B-cell lymphoma (DLBCL) cells, such as miR-125b-5p targeting TNFAIP3, has specifically been demonstrated to reduce sensitivity to rituximab^[29]^; however, the mechanism is unclear. EVs from bone marrow stromal cells (BMSCs) increase resistance to bortezomib, a general cell death activator^[51]^, of MM cells, via promotion of MM homing to the bone marrow and by directly inducing pro-survival signalling pathways^[22]^. Similarly, BMSCs were found to transfer EVs to chronic lymphocytic leukemia (CLL), which promoted resistance to fludarabine, ibrutinib, idelalisib and venetoclax, via upregulation of pro-survival genes and increased migration^[52]^. The transfer of acid sphingomyelinase between MM cells by EVs can also mediate drug resistance to melphalan or bortezomib via an unknown mechanism^[53]^. DLBCL-derived EVs can carry carbonic anhydrase 1 (CA1) that fosters resistance to standard R-CHOP (cyclophosphamide, doxorubicin, vincristine, and prednisone, with rituximab) therapy^[30]^. T-acute lymphoblastic leukemia cells directly transport the multidrug-resistant proteins MRP1 and P-glycoprotein (P-gp) via EVs. However, to the best of our knowledge, this phenomenon has not yet been reported for B cell malignancies. As mentioned above, EVs from lymphoma cells enhance CSR by antagonizing miR-5099 to activate ROD1, which in turn activates AID^[15]^. CSR is associated with two rounds of proliferation and expression of switched immunoglobulins in stronger signals from the BCR, which would further promote survival and proliferation^[54]^. These studies clearly show that both cancer-derived and stromal cell-derived EV proteins and nucleic acids can directly promote proliferation and/or resistance to chemotherapy.

### EVs alter the tumor microenvironment to indirectly support tumor progression

Indirectly, EVs can influence immune system function to promote B cell cancer progression [Figure 2]. For example, DLBCL-derived EVs promote M2 polarization of macrophages by increasing the expression of functional PPARG coactivator 1 beta (PGC-1β) protein, which in turn promotes tumor progression^[25]^. M2 macrophages secrete various cytokines such as transforming growth factor-β (TGF-β) and interleukin-10 (IL-10), which suppress the anti-tumor activity of T cells and Natural Killer (NK) cells and, thus, promote tumor cell growth^[55,56]^. B lymphoblastoid cell-derived EVs carry FASL molecules, which cause CD4^+^ T cells to undergo apoptosis through FAS and FASL, to diminish the anti-tumor response^[57]^. Furthermore, EVs from MM can decrease the levels of perforin secreted by CD8^+^ T cells and increase the viability of Treg cells isolated from healthy donors^[58]^. CD19^+^ EVs originating from normal B cells contain high levels of CD39 and CD73, enzymes that hydrolyze the adenosine triphosphate (ATP) released by chemotherapy-treated tumor cells into adenosine^[59]^. The increase in adenosine can then attenuate the post-chemotherapeutic anti-tumor CD8^+^ T cell responses towards solid tumors. B cells were also found in this study to increase EV production via HIF-1α-mediated upregulation of Rab27a, a key regulator of EV secretion. Conversely, targeted inhibition of Rab27a in B cells restored CD8^+^ T cell function and enhanced chemotherapy efficacy. Moreover, bone marrow stromal cell-derived EVs in patients with MM modulate anti-tumor immunity by promoting the survival of myeloid-derived suppressor cells (MDSCs), which are immunosuppressive^[22]^. Thus, EVs derived directly from malignant or normal B cells, or from the microenvironment, can suppress the anti-tumor response.

EVs can alter the microenvironment to promote tumor cell survival. Exosomes derived from CLL cells can transform stromal cells into cancer-associated fibroblasts (CAFs). CAFs secrete increased levels of pro-inflammatory and pro-survival cytokines (e.g., IL-8, BAFF, CXCL1, LIF, IL-6, and others), reshaping the microenvironment to indirectly support cancer cell survival and proliferation^[27]^. Similarly, CLL-derived EVs taken up by surrounding endothelial cells cause phosphorylation of STAT3, which increases IL-6 secretion from the endothelial cells to create an immunosuppressive environment. Furthermore, STAT3 activation significantly decreases apoptosis in the endothelial cells, further promoting a protective niche for tumor cells^[26]^. EVs derived from MM can also inhibit osteoblastic differentiation and enhance IL-6 secretion from BMSCs, which also contributes to the immunosuppressive and tumor-supportive microenvironment^[28]^. EVs from B lymphoma cells carry CD20, which acts as a decoy by binding to the anti-CD20 therapy rituximab to reduce efficacy of the immunotherapy^[60]^. Interestingly, the drug-efflux protein ATP-binding cassette (ABC) transporter A3 (ABCA3) is essential for the secretion of these EVs from B lymphoma cells^[60]^. Overall, EVs derived from B cell malignancies can support cancer cell survival by modulating the tumor microenvironment via multiple mechanisms [Figure 2].

EVs from solid tumor cells or cells in the tumor microenvironment can also directly affect normal B cell function. In glioblastoma, tumor-infiltrating MDSCs release EVs that can promote the differentiation of mature naïve B cells into B regulatory (Breg) cells (both mouse and human), which were immunosuppressive^[31]^. This differentiation was due to the transfer of PD-L1 from MDSCs to B cells via the EVs. PD-L1^hi^ Bregs suppressed CD8+ T cell activation, however, it was not determined if this was directly via PD-L1/PD-1 interactions or via IL-10 and TGF-β secretion. Similarly, EVs in plasma from patients with esophageal squamous cell carcinoma were able to induce differentiation of circulating CD19+ B cells into IL-10 and PD-L1 expressing Breg cells; however, the ability of these cells to suppress T cell activation was not reported^[32]^. EVs from hepatocellular carcinoma cell lines were able to induce differentiation of CD19+ human B cells into Breg cells via the release of HMGB1^[33]^. These cells, which were TIM-1+, suppressed CD8+ T cells but the contribution of PD-L1, IL-10 or TGF-β to this process is not known. More work is needed to determine whether EVs from solid tumors commonly induce Breg differentiation or if the examples reported above are isolated cases.

### B cell EVs have anti-tumor roles

Studies have also shown that B cell-derived EVs can have anti-tumor effects. CLL B cells activated via CD40 and IL-4 release EVs enriched in miR-363, which are subsequently taken up by CD4+ T cells^[23]^. Uptake of these EVs into T cells caused a downregulation of early T cell activation regulator CD69 expression, which leads to increased migration, immune synapse formation, and interaction of CD4^+^ T cells with tumor cells. B cell-derived EVs have MHC-I and -II molecules on their surface for antigen presentation^[61]^. In this context, EVs secreted from B cells that contain abundant MHC-II molecules have been shown to stimulate antigen-specific CD4^+^ T cell responses *in vitro*^[9]^. A similar response *in vivo* would promote a T cell response against the malignant B cells to promote their apoptosis. Moreover, B cell-derived EVs can transport complement 3 (C3) fragments that interact with G protein coupled receptors on T cells^[62]^. This interaction promotes better T cell responses even when antigen concentration is suboptimal, thereby further increasing the anti-tumor immune response. Thus, while most of the literature to date supports a pro-tumorigenic role for EVs in B cell malignancies, they may also be able to augment the anti-tumor immune response. Therefore, therapeutics that target EVs should be designed to specifically inhibit pro-tumorigenic functions while allowing EVs to be involved in immune activation. For example, therapeutics that target global EV release would also inhibit the anti-tumor immune response.

The context-dependent role of EVs in malignancy partially mirrors the context-dependency seen in normal B cell development. In both cases, under basal conditions EVs suppress the activation of normal immune cells or stromal cells to alter the microenvironment. In contrast, EVs can interact with T cells to promote further immune activation. This apparent paradox reflects the plasticity of EV cargo, which is dependent on many factors including the microenvironment. Therefore, one research priority is elucidating the molecular mechanisms regulating EV cargo sorting and secretion. Improving our understanding of these mechanisms could help develop strategies to selectively inhibit immunosuppressive and resistance-promoting vesicles while allowing immunostimulatory EVs to maintain their function.

## EVs AS BIOMARKERS FOR THE DETECTION OF B CELL MALIGNANCIES

B cell-derived cancers can be detected through a blood draw when the cancer cells are highly abundant and circulating. However, in early stages of disease or after treatment, the cancer cells are not readily accessible through peripheral blood draws. Therefore, clinicians must perform invasive biopsies such as bone marrow aspirations or spinal biopsies, particularly in the case of leukemia and MM where imaging scans are non-informative. After treatment, any remaining cancer cells are termed minimal (or measurable) residual disease (MRD). MRD in B cell cancers is measured using highly sensitive assays such as flow cytometry or polymerase chain reaction (PCR). Multiparameter flow cytometry, for example, can detect one cancer cell in 1,000 to 10,000 normal bone marrow cells in bone marrow aspirates^[63]^. However, these invasive procedures can be frightening and uncomfortable, particularly for pediatric patients and carry a risk of infection. Thus, discovering a better method of detecting B cell malignancies is valuable.

Cancer cells tend to secrete more EVs than normal cells, which is reflected by the increased concentration of EVs found in the plasma of cancer patients in many different types of cancers originating from B cells, including DLCBL^[64-67]^, CLL^[64,68,69]^, MM^[64,70]^, B lymphoblastic leukeumia (B-ALL)^[64,71-74]^, and Waldenström macroglobulinemia^[64]^. In fact, this increase is by far the most consistent finding in the studies we reviewed to date. This increase in EV concentration independently predicts overall survival in CLL^[69]^, and likely other B cell cancers. One exception to this trend is when acetylcholinesterase activity was used to quantify EVs^[75]^, which has since been found to be an unreliable marker^[76]^.

### Potential protein biomarkers

In B-ALL, there was a specific increase in EVs expressing EV markers (CD9, CD63), B cell-specific antigens [CD19, CD34, human leukocyte antigen (HLA)- DR], cell adhesion molecules (CD146), and stem cell antigens (CD44, CD105)^[74]^. After treatment, the concentration of EVs and/or EV cargo decreased. In the case of B-ALL, EVs expressing CD10 and CD19 were found to decrease 15 days after induction therapy and remained reduced 35 days after treatment^[72]^. In addition, EV-specific miR-128-3p expression levels decrease during the month after induction therapy in B-ALL patients^[77]^. These changes were strongly predictive of MRD as measured by flow cytometry 15 days after induction. In the case of acquired immunodeficiency syndrome (AIDS)-related non-Hodgkin’s lymphoma, which includes Burkitt’s lymphoma and DLBCL, there was a specific reduction in EVs expressing PD-L1, CD40, CD40-ligand (CD40L), or tumour necrosis factor receptor II (TNF-RII) after treatment with rituximab and/or combination chemotherapy^[78]^. In DLBCL, an increase in specific detection of CD9^+^CD63^+^ and PD-L1^+^CD63^+^ EVs at diagnosis was associated with worse survival^[65]^. However, in all cases, there was a variety of responses and an overlap between patients with disease compared to healthy controls in many cases, thus highlighting the need for biomarker signatures, rather than single gene or protein diagnostic test.

Additional studies have taken non-biased proteomics approaches to identify disease-specific signatures for B cell neoplasia [Table 2]. For example, using shotgun proteomics on isolated EVs, Saidu *et al*. identified a core signature of six proteins present in EVs from B-ALL plasma samples and conditioned media but not in control samples^[73]^. Of these six, PCNA was also validated by western blot while IGF2BP1 was not statistically different. Using a similar approach, 11 proteins were found to be altered in EVs from relapsed to non-relapsed Hodgkin’s Lymphoma (5 decreased, 6 increased) by 2D gel and 161 differentially packaged proteins by shotgun liquid chromatography (LC)- Mass Spectrometry (MS)/MS^[79]^. In another study of DLBCL, the EV proteome was more diverse compared to healthy donors and a very large number (365) of proteins were found to be exclusively in DLBCL plasma EVs compared to healthy controls^[66]^. Using a regression analysis, the authors found a perfect correlation between changes in the top protein and disease prediction. The top five hits of importance to the model are included in Table 2 along with significantly changed proteins that matched findings from other studies. Interestingly, these top hits do not match those that predicted survival, suggesting that biomarkers for diagnosis differ from biomarkers for prognosis. It is unclear what this difference would mean for clinical applicability but, at the very least, it would make any testing more complicated. Regardless, there was very little consensus between studies, highlighting the need for additional studies with increased sample sizes.

**Table 2:** Potential EV biomarkers found in blood plasma EVs

**Cancer type**	**EV biomarker(s)**	**Change**	**Function/Pathway**	**Method (Cohort)**	**Clinical potential**
B-ALL	CD137, CD38, IFG2BP1, PCNA, CSDE1, GPR116	↑ *vs*. healthy	Oncogenic proteins linked to proliferation	LC-MS/MS^a^ (14 BCP-ALL, 18 healthy)^[73]^	Diagnostic; therapy monitoring
B-ALL	CD9, CD63, CD81, CD19, CD34, HLA-DR, CD29, CD146, CD44, CD105	↑ *vs*. healthy	EV surface signature	Bead-based flow cytometry (3 B-ALL, 7 healthy)^[74]^	Prognostic biomarkers
B-ALL	miR-758-3p, miR-335-5p, miR-26b-5p, miR-340-3p, let-7f-5p	↓ *vs*. donors	Tumor suppressor miRNAs	RNA-seq (8 ALL, 6 donors)^[71]^	Prognostic biomarkers
Hodgkin lymphoma (HL)	ORM1, TTR, APOA1, CLU, APOA4, HP	↑ in relapsed HL	Acute-phase proteins, lipid metabolism	2D-DIGE^a^ + LC-MS/MS (6 relapsed, 10 non-relapsed)^[79]^	Predict relapse
Hodgkin lymphoma (HL)	ITIH2, C4B, C4A, FGG, IGHM	↓ in relapsed HL	Complement & immune regulation	2D-DIGE + LC-MS/MS (same as above)^[80]^	Prognostic biomarkers
DLBCL	ORM1, Igκ chain, GNAI3, RAB1B	↑ in DLBCL	Oncogenic signaling proteins	LC-MS/MS (32 DLBCL, 15 controls)^[66]^	Diagnostic; therapeutic targeting
DLBCL	FGG, APOA4, SERPINF1, VNN1	↓ in DLBCL	Coagulation/metabolism	LC-MS/MS (32 DLBCL, 15 controls)^[66]^	Diagnostic biomarkers
DLBCL	miR-124, miR-532-5p	↑ *vs*. healthy	OncomiRs; cell cycle	RNA-seq (24 DLBCL, 20 controls^[80]^	Diagnostic biomarkers
DLBCL	miR-4476, miR-379-5p, miR-135a-3p	↑ *vs*. healthy	Diverse oncogenic miRNAs	Microarray (10 DLBCL, 5 controls^[81]^	Diagnostic biomarkers
DLBCL	miR-181a-5p	↓ *vs*. healthy	Regulates BCR/TCR signaling	RT-qPCR (33 DLBCL, 22 controls)^[82]^	Diagnostic biomarker
DLBCL	miR-451a, miR-483-3p	↓ *vs*. healthy	Tumor suppressors	Microarray (10 DLBCL, 5 controls)^[81]^	Prognostic biomarkers
CLL	miR-150, miR-155, miR-29a/b/c	↑ *vs*. healthy	Known oncomiRs; immune regulation	nCounter + RT-qPCR (69 CLL, 15 controls)^[68]^	Prognostic; immune dysfunction marker
CLL	miR-579, miR-191, miR-302d, miR-223, let-7d, miR-1246	↓ *vs*. healthy	Tumor suppressors	nCounter + RT-qPCR (69 CLL, 15 controls)^[68]^	Prognostic biomarkers
CLL	hY4 (small RNA)	↑ *vs*. healthy	Immune modulation	Northern blot (3 CLL, 3 healthy)^[83]^	Diagnostic biomarker

↑ Indicates increased expression; ↓ indicates decreased expression. ^a^LC-MS/MS: Liquid chromatography-tandem mass spectrometry; 2D-DIGE: two-dimensional difference gel electrophoresis; RT-qPCR: reverse transcriptase quantitative polymerase chain reaction; ALL: acute lymphoblastic leukemia; APOA1: apolipoprotein A1; APOA4: apolipoprotein A4; CD: cluster of differentiation; CLU: clusterin; CLL: chronic lymphocytic leukemia; CSDE1: cold shock domain containing E1; EVs: extracellular vesicles; FGG: fibrinogen gamma chain; GNAI3: G-protein subunit alpha i3; GPR116: G-protein coupled receptor 116; HLA-DR: human leukocyte antigen-DR; HP: haptoglobin; IGHM: immunoglobulin heavy constant mu; IFG2BP1: insulin-like growth factor 2 mRNA-binding protein 1; miR/miRNA: microRNA; ORM1: orosomucoid 1; PCNA: proliferating cell nuclear antigen; SERPINF1: serpin family F member 1; TTR: transthyretin; VNN1: vanin 1.

### Potential RNA biomarkers

Identification of discriminatory small RNA from blood-derived EVs has also been a major focus. Interestingly, one study found that the levels of miRNAs in EVs from the plasma of patients with DLBCL were identical to those isolated from whole serum^[80]^, suggesting that many studies exploring circulating miRNAs may actually be analyzing EV-associated miRNAs. However, we have focused here on studies that have specifically evaluated EV-associated small RNA [Table 2]. Using miRNA expression in DLBCL cells, Liu *et al*. found that miR-107 was downregulated in cells and that this was reflected in EVs^[84]^. miR-107 is predicted to downregulate PI3K, Hippo, and AMPK signalling pathways; thus, its decreased expression is associated with an increase in pro-survival and pro-proliferative signalling. At least two studies have found miRNA signatures that discriminate DLBCL from healthy donors^[81,82]^, but they did not share a single miRNA in their signatures nor did they identify miR-107. Notably, the studies used different methods to identify the miRNAs [i.e., reverse transcriptase quantitative (RT-qPCR) *vs*. microarray]. For CLL, a handful of miRNAs were found to be altered in CLL patient samples^[68]^, as well as an increase in hY4 small RNA was observed^[83]^. In B-ALL, a small RNA signature clearly distinguished pediatric patients from children without leukemia but surprisingly the majority of the discriminatory genes were mRNA fragments rather than miRNAs^[71]^. Therefore, although each individual study showed promise for a diagnostic and/or prognostic test, there was very little agreement between studies. This could be because the methods for EV isolation and characterization differed or because of small sample sizes. There are also only a limited number of studies available, so generalization is not possible. Overall, this highlights the need for additional work to be done to characterize the RNA EV cargo in relevant biological samples.

While the majority of studies describe the identification of small RNAs, there is evidence that longer RNAs may be packaged intact. However, most studies of mRNAs in EVs employed RT-qPCR, which generated amplicons of < 150 bases that would not be distinguishable from fragments of full-length mRNAs^[71]^. In our case, using short-read RNA sequencing (RNA-seq), we found that in some cases reads from across the transcript were sequenced; however, more work must be done to discriminate between sequencing fragments and actual fragments before firm conclusions can be made. Encouragingly, delivery of DNMT1 mRNA from pediatric ALL and multiple transcripts from CLL EVs into recipient cells has been described with subsequent protein translation shown for the TCL1A protein from CLL EVs^[85,86]^.

### Challenges using EVs as biomarkers

There are major challenges with using EVs as biomarkers starting with collection and pre-processing through to EV isolation and marker detection. Appropriate collection is essential as platelet activation within the collection tube can result in contamination by platelet EVs. Timing is an important consideration and blood samples should be processed within 30 min if collected in Ethylenediaminetetraacetic acid (EDTA) tubes (unpublished observations). Development of stabilizing agents (e.g., STREK tubes) can increase the time before processing by preventing platelet activation and cell death-mediated release of EVs and nucleic acids; however, this is limited to 8 h or less^[87]^. In addition, two additional centrifugations prior to storage are recommended to generate platelet-depleted plasma, another factor that may vary between sources. With respect to EV isolation, contamination by other plasma components such as lipoproteins and/or protein aggregates is common with ultracentrifugation (UC), size-exclusion chromatography (SEC), density-based isolations, and precipitation approaches^[88]^. Using multiple methods adds specificity to the isolation but adds time and there may be a concomitant loss of EVs. Immuno- or peptide-affinity approaches, on the other hand, have increased specificity but may risk losing some informative EV sub-populations. Our group has favored a peptide-affinity approach (Vn96) based on interactions with heat shock proteins (HSP)^[89]^, which pulls down multiple EV sub-populations^[71]^; however, protein and cell-free DNA are potential contaminants^[90]^. It is, therefore, critical that EV isolation methods are fully documented to allow proper comparison between studies as recommended in the MISEV guidelines^[6]^. In addition, we have found that different RNA isolation kits are not equivalent for detection of small RNA transcripts by RNA-seq (unpublished observations). Lastly, there have been significant advances in technology for the detection of protein or RNA cargo. Microarray or RT-qPCR were common practices for identification of RNA cargo in the past; However, with the advent of affordable next-generation sequencing (NGS), full, unbiased analysis of cargo by RNA-seq should become standard. Similarly, improvements in proteomics allow for non-biased protein detection. Unfortunately, technical differences often make cross-comparisons impossible. For EV-based biomarker analysis to be clinically useful, either isolation techniques need to be standardized or a biomarker signature needs to be identified that is robust enough to be detected regardless of the isolation method. We personally favor this latter goal as this will allow flexibility in clinical diagnostic labs to choose the protocol that is most compatible with their process. Future studies should therefore attempt to find signatures common to multiple isolation techniques.

## OUTLOOK AND FUTURE DIRECTIONS

A number of knowledge gaps remain, both for the fundamental understanding of EVs in normal and malignant B cell development and activation as well as in the translational potential for EVs. Fundamentally, the differing roles that EVs appear to play in the bone marrow microenvironment compared to secondary lymphoid organs need to be investigated more thoroughly. It is unclear if EVs from B cells or MSCs truly influence B cell development in the bone marrow *in vivo* and which stage(s) of B cell development are affected. In addition, determining how EVs from Mast cells induce isotype switching may lend insight into allergy prevention strategies to interrupt the positive feedback loop created by the EVs. Understanding the contribution of EV-bound antibodies to infection control compared with unbound circulating antibodies will provide additional insights into immune system function. It is also important to understand the mechanisms underlying the selective sorting of key cargo into EVs from malignant B cells compared to those from normal B cells. This would allow for selective targeting of EVs that are promoting the malignancy while maintaining or stimulating the immune-activating function of EVs.

With respect to the translational potential of EVs, more studies on EV cargo - both protein and RNA - are needed before EVs can be used as clinical biomarkers. This requires cross-validation using more than one isolation method, as well as larger sample sizes than those currently reported in the literature. Collection of longitudinal plasma samples will also be extremely important for these analyses, although this is not typically done in blood cancer-focused biobanks based on our experience. Moving toward EVs as biomarkers may enable more sensitive and more frequent detection of disease, potentially leading to better treatment timing.

EVs are also attractive as therapeutic vehicles because they are stable yet often non-immunogenic. However, before they can be used as therapies, much more work needs to be done on understanding how EV uptake is regulated with respect to target cell specificity and uptake mechanisms. Moreover, understanding how RNA or protein is selectively packaged will allow researchers to selectively load key cargo into EVs while understanding how EV release is regulated could help with EV recovery *in vitro* or in the development of *in situ* therapies to modify EV release.

## SUMMARY

The evidence published to date supports the role of B cell-derived EVs as modulators of immune function and cancer progression, but their influence appears highly contextual. Under homeostatic conditions, they tend to be immunosuppressive, whereas under immune activation, EVs further promote B cell responses. In cancer, the impact of EVs on anti-tumor immunity is predominantly indirect, mediated through modulation of the tumor microenvironment or activation of immune suppressor cells. Therapeutically, targeting EV-mediated mechanisms, either by inhibiting EV formation, release, or uptake, represents a promising approach to overcoming drug resistance but needs to be approached cautiously as this may also impact the immune-activating function of EVs. Continued advanced characterization of EV uptake pathways could potentially enhance the effectiveness of cancer treatments as EVs could be used as non-inflammatory drug-delivery vehicles^[22,91,92]^. The use of EVs as adjuvants to enhance immune activation is also a promising but underexplored area. Moreover, B cell-derived EVs could be used as biomarkers to monitor disease progression once clear, robust signatures are identified. Overall, EVs should remain a key research priority due to their considerable potential for multiple clinical applications.

## DECLARATIONS

### Copyright

© The Author(s) 2025.
